# Intralesional steroid injection versus voice therapy for the management of early vocal fold nodules in female patients: a randomized controlled trial

**DOI:** 10.1007/s00405-025-09923-7

**Published:** 2025-12-29

**Authors:** Eman S. Hassan, Nada A. Kamel, Hanan A. Mohamed

**Affiliations:** Phoniatric Unit, ENT Department, Faculty of Medicine, Assiut University, Assiut, 71516 Egypt

**Keywords:** Vocal fold nodules, Management, Steroid injection, Triamcinolone, Accent method, Voice therapy

## Abstract

**Purpose:**

While vocal fold steroid injection (VFSI) is emerging as a therapeutic approach for vocal fold nodules (VFNs), its comparative efficacy relative to standard voice therapy remains unestablished. This study aimed to evaluate and contrast treatment findings between VFSI and voice therapy for VFNs.

**Methods:**

In a randomized controlled trial, 50 female patients with VFNs received either percutaneous intralesional triamcinolone injection or Accent method voice therapy. Serial evaluations—including videoendoscopic (nodule size, mucosal wave, glottic gap), subjective (auditory perceptual assessment, Voice Handicap Index), and objective (acoustic, aerodynamic) measures—were conducted at baseline and at 1 week and 1-, 2-, 3-, and 6-months post-treatment.

**Results:**

Both groups demonstrated significant reductions in nodule size and improvements in mucosal wave patterns and glottic gaps over the 6 months (*p* < 0.05). Most subjective and objective measures also enhanced significantly in both groups over the same study period (*p* < 0.05). However, voice therapy outperformed VFSI in most parameters (videoendoscopic, subjective, and objective) by the end of the third month (*p* < 0.05) and in nodule size reduction by the sixth month (*p* = 0.049).

**Conclusions:**

VFSI is a rapid and reliable therapeutic solution for VFNs with outcomes comparable to voice therapy. Long-term data, however, demonstrate voice therapy better prevents nodule recurrence. Therefore, subsequent voice therapy or repeated injections may enhance the long-term efficacy of VFSI.

## Introduction

Vocal nodules represent benign lesions that commonly develop bilaterally, albeit not necessarily symmetrically, within the lamina propria layer of the vocal folds (VF) [1]. They constitute the most prevalent form of organic laryngeal pathology among professional voice users and voice patients more broadly, being diagnosed in approximately 15% of individuals presenting with dysphonia [2]. They result from chronic vocal abuse or misuse and frequently manifest in children and adult females, especially those in occupations related to voice [3]. Repeated trauma to the mid-membranous VF segment induces stromal edema, vascular dilatation, and fibroblast proliferation [4].

Their presence affects the VF vibratory pattern and adds to the VF mass. Also, it disrupts the glottic closure anteriorly and posteriorly to the nodule [5]. The resulting dysphonia is strained, breathy, and rough, with varying degrees of turbulent noise and a low-pitch tendency [5–7]. These changes can lead to occupational, social, and personal problems [8, 9].

Conservative management—particularly voice therapy—represents the first-line intervention. Among the holistic approaches to vocal behavior modification, the Accent Method stands out by addressing multiple vocal parameters, including pitch, loudness, and timbre [10]. Nevertheless, adherence to voice rest and adequate voice therapy are sometimes challenging to implement in individuals with vocally demanding professions [11, 12]. In cases refractory to conservative therapy, laryngeal microsurgical resection under general anesthesia is indicated; nevertheless, the function of surgical management is significantly limited [13].

The clinical use of steroids in otolaryngology leverages their potent anti-edema and anti-inflammatory properties. Steroids impede wound healing through suppressed collagen synthesis, altered wound stiffness, inhibited fibroblast activity, and diminished bactericidal and phagocytic functions of specific defense cells [14]. These actions are theorized to directly address the vocal nodule pathophysiology. Thus, intralesional steroid injection for VFNs may bridge therapeutic space separating behavioral voice treatment and laryngomicroscopic surgical procedures.

Numerous studies have examined VFSI in benign lesions, including nodules [15–20]. Injections were done under local anesthesia by various approaches, including transoral, transnasal, and percutaneous. In two studies concerned only with vocal nodules, laryngoscopic evaluation revealed that 93–100% of nodules either regressed or fully resolved after nearly 1 month of VFSI [12, 21]. The documented symptom/nodule recurrence rate after two years was 31% [22]. Injectable steroids that were described include dexamethasone sodium phosphate [19, 23], triamcinolone acetonide [12, 17, 20, 21, 24], a 1:1 mixture of triamcinolone acetonide and dexamethasone sodium phosphate [18, 22], methylprednisolone acetate [16, 25], and betamethasone sodium phosphate [26]. To the best of our knowledge, no prior investigation has conducted a comparative analysis of vocal nodule steroid injection with an active control receiving voice therapy to accurately assess the clinical role of VFSI. Only Wang et al. studied the difference in reduction in VF benign lesion size subsequent to either VFSI or vocal hygiene education (VHE) [23]. They reported that VFSI demonstrated a greater rate of nodule reduction compared to VHE at the 1-month follow-up assessment. This study aims to evaluate the efficacy of VFSI compared to the Accent method of voice therapy in managing VFNs. Additionally, it compares the follow-up results of both treatment modalities to assess their long-term therapeutic effects.

## Methods

This open label randomized controlled trial was performed in the Phoniatric Unit, Assiut University Hospital, Assiut, Egypt. It was carried out between June 2019 and August 2023 following approval by the Assiut Faculty of Medicine Ethics Committee (Code: 17200338) and ClinicalTrials.gov registration (ID: NCT03914092). The study enrolled 50 female patients aged between 18 and 55 years with bilateral soft edematous VF nodules that didn’t exceed 2.5 mm at the base and 0.5 mm at the apex, with preserved or minimally impacted stroboscopic waves. Informed written consent was obtained from all participants prior to the study. Patients with previous voice therapy or microphonosurgery, use of drugs (which may induce alterations in laryngeal function, mucosal integrity, or muscular activity), a history of allergies, pulmonary disorders, or gastroesophageal reflux disease (GERD), as well as the presence of other concomitant vocal fold pathologies—such as vocal polyps, cysts, precancerous or malignant lesions, vocal fold paralysis, scarring, atrophy, or sulcus vocalis, etc.— and current psychiatric or neurologic conditions were excluded.

### Randomization

Computer-generated random numbers were used for patient randomization. Participants were allocated into two equal groups (GPs): GP I (steroid injection GP), with VFNs undergoing office-based percutaneous triamcinolone acetonide VF injection, and GP II (voice therapy GP), with VFNs undergoing Smith Accent voice therapy.

### Sample size calculation

It was calculated grounded in previous findings [20, 27] using the G*Power program version 3.1.9.7 to determine the sample size; it was guided by the difference between two independent proportions using the two-tailed z-test, α error = 0.05, and power (1-β error) = 80%. The total estimated sample size was 38 cases (19 cases per GP) and increased to 50 when adding 25% to account for potential participant attrition.

Both GPs underwent the following assessments before intervention and at the follow-up visits: one week and 1-, 2-, 3-, and 6-months post-intervention completion.

### Patient’s interview (history taking)

Data was collected from recruited patients, including age, onset, course, duration of symptoms, occupation, vocal demand, voice abuse, smoking, education, marital status, and number of children.

### Subjective voice assessment

Subjective evaluation encompassed an auditory-perceptual assessment of voice (APA) and the Voice Handicap Index (VHI).

The APA of each patient was evaluated pre- and post-intervention using the GRBAS scale (Grade of dysphonia, Roughness, Breathiness, Asthenia, and Straining) [28]. The grades were categorized into 0 = Normal, 1 = Mild deviation, 2 = Moderate deviation, and 3 = Severe deviation. Scores from the five subscales were summed up for statistical inferences. Continuous speech samples, each lasting over 40 s and recorded with high fidelity, were independently and blindly evaluated by two expert phoniatricians. The intra- and inter-judge agreement for dysphonia was reported.

The VHI (Arabic version) is a self-assessment questionnaire used to measure patients᾿ perceptions of their voices [29]. It comprises three domains: functional, physical, and emotional. The domain has ten items, each with a score ranging from zero to four (0 = never, 1 = almost never, 2 = sometimes, 3 = almost always, 4 = always). The sum of these domains was calculated.

### Videolaryngoscopic examination (VLS)

**Fig. 1:**
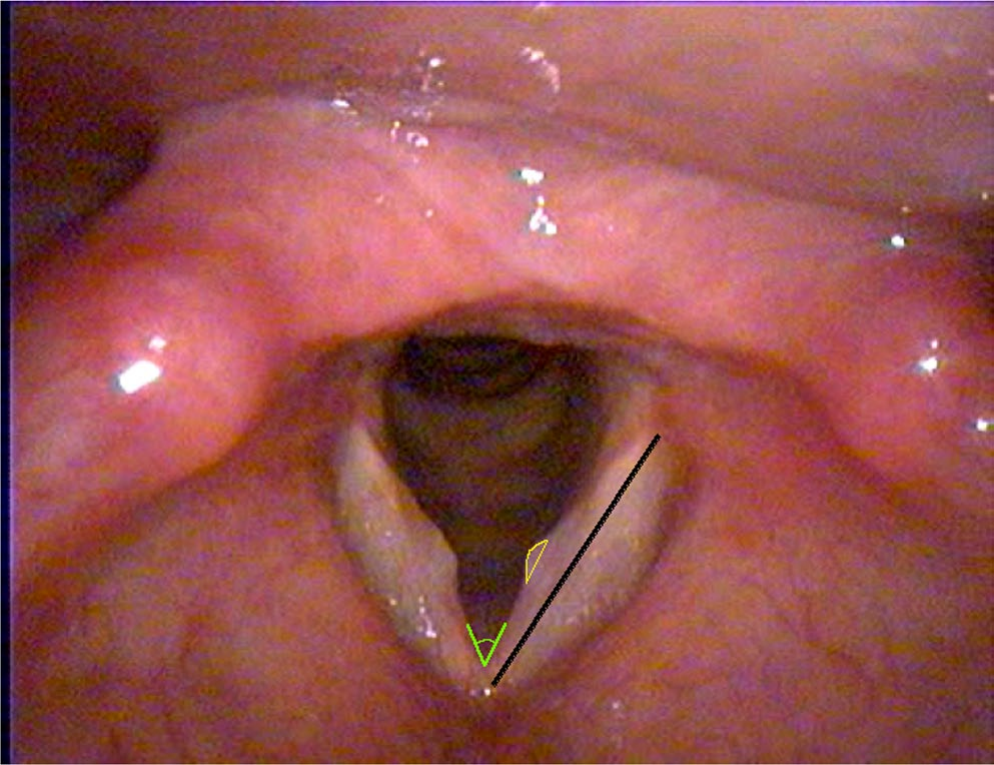
Measurements of nodule size (circumscribed area in yellow), vocal fold length (black line), and open angle of bilateral vocal folds (green lines) using ImageJ software

It was performed using a charged coupled device (CCD) videorhino-laryngoscopy (Tele Pack X LED TP100; Karl Storz, Tuttlingen, Germany) or rigid strobo-laryngoscope with integrated HOPKINS^®^ lateral telescope 70° (Model 8706 CA Karl Storz; Tuttlingen, Germany). A computer hard drive (H0W79EA#ABV HP 250 G1 Notebook PC, Hewlett-Packard, China) has digital recordings of every VLS test session. The following data were obtained:

Measurement of nodule size in pixels was done (Fig. 1). Still images of the vocal nodules were acquired at same dimensions (720 × 557 pixels), and the nodule area was delineated using ImageJ software (version 1.52a, National Institutes of Health, USA). To account for variations in apparent nodule size due to differences in the distance between the endoscope tip and the vocal folds, adjusted nodule sizes were calculated as follows:

$$\:\mathrm{N}\mathrm{o}\mathrm{d}\mathrm{u}\mathrm{l}\mathrm{e}\:\mathrm{s}\mathrm{i}\mathrm{z}\mathrm{e}=\frac{\mathrm{c}\mathrm{i}\mathrm{r}\mathrm{c}\mathrm{u}\mathrm{m}\mathrm{s}\mathrm{c}\mathrm{r}\mathrm{i}\mathrm{b}\mathrm{r}\mathrm{d}\:\mathrm{n}\mathrm{o}\mathrm{d}\mathrm{u}\mathrm{l}\mathrm{e}\:\mathrm{a}\mathrm{r}\mathrm{e}\mathrm{a}\:}{\mathrm{V}\mathrm{F}\:\mathrm{l}\mathrm{e}\mathrm{n}\mathrm{g}\mathrm{t}\mathrm{h}\:}$$

The vocal fold (VF) length was defined as the distance from the anterior commissure to the posterior margin of the vocal process. To ensure consistency in VF length measurements, the inter-VFs angle was assessed; an angle exceeding 40° was deemed adequate for standardization [30].

The vibratory properties of the mucosa and the glottic gap assessments were also conducted and blindly evaluated by two experienced phoniatricians. A 4-point equal-interval scale was employed to assess the mucosal wave [24]: 0 = absent, 1 = severely reduced, 2 = mildly reduced, and 3 = intact. In addition, the glottal gap was assessed using a 4-point equal-appearing interval scale, defined as follows [15]: 0 = severe (>2 mm), 1 = moderate (1–2 mm), 2 = mild (< 1 mm), and 3 = absent. The intra- and inter-judge agreement for both mucosal wave and glottic gap was documented. 

The following side effects were identified by analyzing video recordings taken after VFSI: (1) Vocal fold (VF) hematoma localized at the injection sites; (2) deposition of the injected material, identified as a whitish plaque within Reinke’s space and associated with impairment of mucosal wave propagation; and (3) VF atrophy, characterized by vocal fold bowing, atrophy of the thyroarytenoid muscle, and the presence of a glottic gap.

### Objective voice assessment

Acoustic voice analysis was performed in a quiet, sound-treated room. Patients were instructed to produce a sustained phonation of the vowel /a/ for 3–4 s at a comfortable pitch and loudness. The recorded samples were analyzed using Computerized Speech Lab (CSL) software (model 4300B; Kay Elemetrics Corp., USA) to extract acoustic parameters, including fundamental frequency (F0, in Hz), jitter (in %), shimmer (in dB), and harmonic-to-noise ratio (H/N, in dB).

Maximum phonation time (MPT) is an aerodynamic parameter, expressed in seconds, determined by timing the sustained production of the vowel /a/ at a comfortable pitch and intensity, using an audio recording device and a stopwatch.

### Intervention techniques

#### Vocal fold steroid injection (VFSI) (1st GP)

Before VFSI, anesthetization of the patient’s nose, oral cavity, tonsils, vallecula, and pharynx with 10% lidocaine spray and sterilization of the skin overlying the cricothyroid membrane were done. A CCD videorhinolaryngoscopy was used to administer the injection, with the help of an experienced assistant. The surgeon then injected 5 ml of lidocaine hydrochloride 2% directly into the cricothyroid membrane. A 23-gauge × 1 ¼ inch needle on a 3-mL disposable plastic syringe was bent at its base 45° after being filled with 0.1–0.3 mL of triamcinolone acetonide (40 mg/mL) [31]. It was then introduced through the cricothyroid membrane, and the needle was positioned superomedially. Video monitoring confirmed needle tip positioning in the subepithelial space, followed by slow infusion of the injected suspension, avoiding deep infusion into the vocalis muscle. The whole procedure took from 15 to 30 min.

All participants received the injection bilaterally, with one uncooperative patient whose left vocal nodule failed to be injected. Participants were administered a one-time injection with no subsequent doses and were provided with instructions not to phonate for two days to prevent material leakage and increase absorption at nodules.

#### The accent method of voice therapy (AM) (2nd GP)

Patients in the second GP received 24 regular individual sessions of Smith Accent voice therapy, administrated twice weekly over a three-month period, with each session lasting 20 min. It was started by establishing rapport, then preparing for breathing exercises, followed by abdomino-diaphragmatic breathing exercises, then phonatory exercises with accentuated vowel play at different rhythms (largo, andante, and allegro) linked then with body and arm movements, and finally stabilization of the newly acquired vocal behavior to spontaneous dialogue was elicited through a series of tasks with progressively increasing difficulty, including (a) articulated vocal play using nonsensical syllables, (b) automatic speech, (c) repetition of utterances produced by the therapist, (d) reading aloud, and (e) monologue production [32].

Patients in both GPs received instruction on VHE during the initial clinical visit, as outlined in a printed document that was given to them. VHE instructions included phonation habit modification, workplace microphone amplification, proper hydration, minimizing throat clearing, and securing adequate vocal rest.

### Statistical analysis

Statistical Package for Social Science (SPSS version 27.0 for Windows, IBM Corp., Armonk, New York) was applied for data management and analysis. Mean and standard deviation were used to describe quantitative data, while qualitative data were represented as numbers with percentages. The following tests were conducted:

Pre- and post-intervention comparison: Friedman’s test compared more than two intervals in each studied GP (between pre- and 1 week, 1-, 2-, and 3-months post-intervention). While the Wilcoxon signed-rank test was used to compare pre- and post-6-month voice parameters.

Between-GPs comparison: The Chi-square (for parametric data) and Fisher’s Exact tests (for non-parametric data) were employed to compare independent categorical variables. While the independent T-test (for parametric data) and the Mann-Whitney test (for non-parametric data) were applied to analyze relative to continuous variables. Intra- and inter-rater agreement: Intra-rater reliability for mucosal wave, glottic gap, and GRBAS was calculated using Cronbach’s Alpha (Guttman Split-Half Coefficient), while inter-rater reliability was assessed by Chi-Square tests with Kappa agreement. A P-value < 0.05 was considered statistically significant.

## Results

**Fig. 2:**
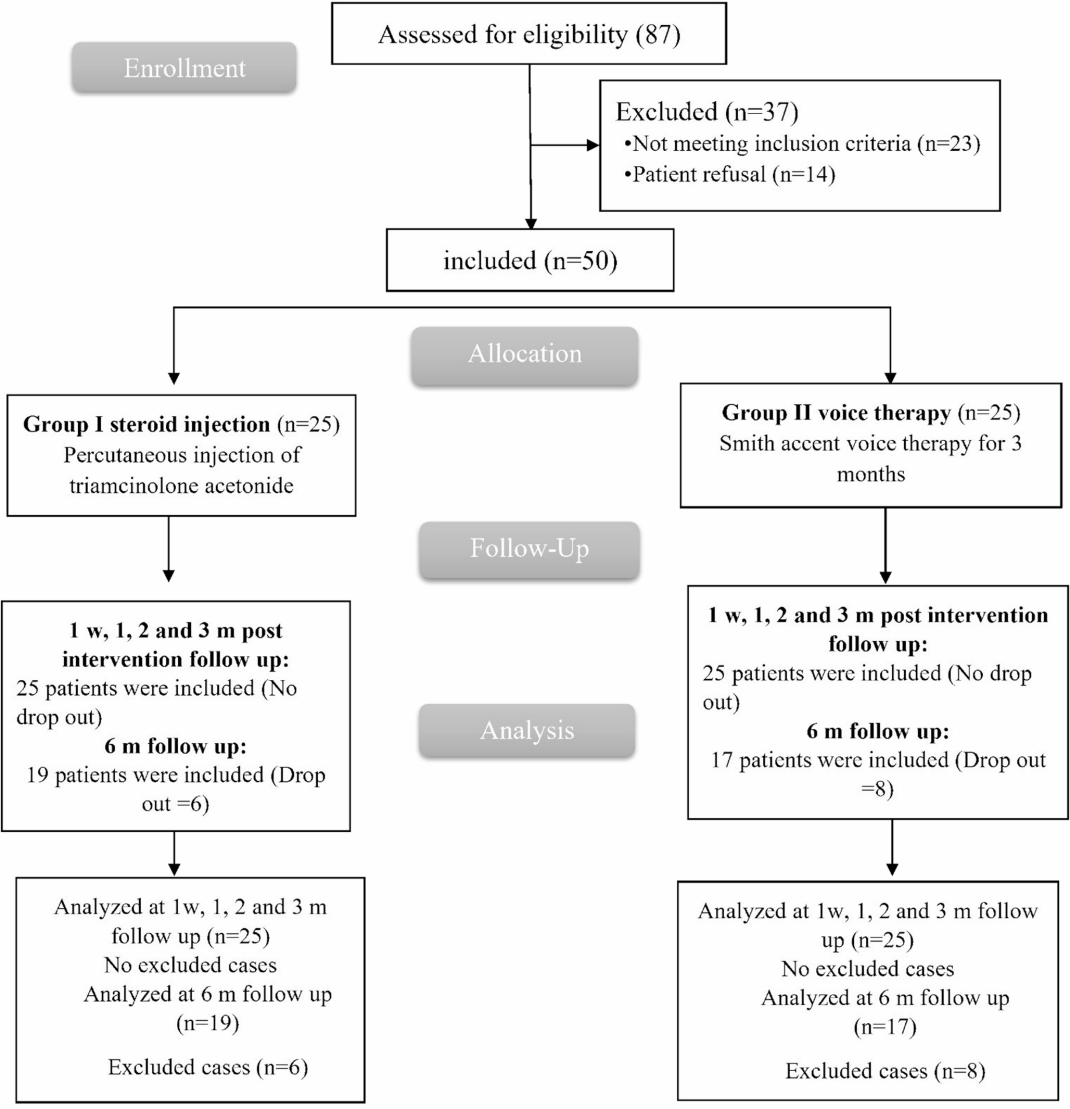
CONSORT flowchart of the enrolled patients

Out of 87 female participants, 23 did not meet eligibility criteria, and 14 declined. Fifty were randomly divided into two GPs, followed up, and analyzed for 3 and 6 months, excluding those who dropped out (Fig. 2).

There was no statistically significant difference between the demographic and clinical characteristics of the patients in the two GPs (Table 1; *P* > 0.05).

**Table 1:** Demographic and clinical characteristics of studied patients

Characteristic	Category/Measurement	GP Ⅰ (*N* = 25)	GP ⅠⅠ (*N* = 25)	*P*-value
Age (years)	-	36 ± 9	35 ± 9	0.949
Symptom duration (months)	-	24.6 ± 23.1	24.3 ± 27.8	0.641
Marital status	Single	1 (4.0%)	2 (8.0%)	0.103
	Married	20 (80.0%)	23 (92.0%)	
	Divorced	4 (16.0%)	0 (0.0%)	
Number of children	-	3 ± 2	3 ± 1	0.215
Occupation	Housewife	16 (64.0%)	12 (48.0%)	0.208
	Kindergarten Nanny	1 (4.0%)	0 (0.0%)	
	Lawyer	0 (0.0%)	1 (4.0%)	
	Nurse	0 (0.0%)	2 (8.0%)	
	Quran Tutor	3 (12.0%)	1 (4.0%)	
	Security Officer	1 (4.0%)	0 (0.0%)	
	Student	0 (0.0%)	2 (8.0%)	
	Teacher	4 (16.0%)	7 (28.0%)	
Occupational vocal demand	Ordinary	2 (8.0%)	4 (16.0%)	0.435
	High	20 (80.0%)	20 (80.0%)	
	Professional	3 (12.0%)	1 (4.0%)	
Temper	Quite	4 (16.0%)	2 (8.0%)	0.384
	Tense	21 (84.0%)	23 (92.0%)	
Voice abuse	Negative	6 (24.0%)	4 (16.0%)	0.480
	Positive	19 (76.0%)	21 (84.0%)	
Smoking	Negative	16 (64.0%)	21 (84.0%)	0.107
	Passive	9 (36.0%)	4 (16.0%)	
Education	High	11 (44.0%)	12 (48.0%)	0.934
	Secondary	7 (28.0%)	6 (24.0%)	
	Primary	3 (12.0%)	2 (8.0%)	
	Illiterate	4 (16.0%)	5 (20.0%)	

### Overall treatment effects over time

Nodule size was significantly lower, and mucosal wave and glottic gap were markedly higher at 1 week and 1-, 2-, and 3-months post-treatment in both GPs, indicating improvement (P value < 0.001). The inter-judge agreement of mucosal waves across the pre- and 3-month follow-up ranged from substantial to perfect agreements (kappa agreement: 0.721 to 0.929, *p* < 0.001) and perfect agreement for glottic gap (kappa agreement: 0.841 to 0.908, *p* < 0.001). Meanwhile, the intra-rater reliability for mucosal wave and glottic gap was 0.983 and 0.993, respectively. The total scores of GRBAS and VHI were significantly lower over the entire 3 months post-treatment in both GPs (P value < 0.001). The inter-judge agreement of GRBAS across the pre- and 3-month follow-up showed perfect agreements (kappa agreement: 0.846 to 0.896, *p* < 0.001), and intra-rater reliability of 0.993.

F0 in the steroid injection GP was significantly higher (P value = 0.006), whereas it was significantly lower in the voice therapy GP over a 3-month follow-up (P value < 0.001). Jitter and shimmer were markedly lower, while the H/N ratio and MPT were elevated at 3 months after intervention in both GPs (P value < 0.001) (Table 2).

**Table 2:** Comparison of endoscopic assessment, subjective and objective parameters pre and 3-month post-treatment in both GPs

		GP I (*N* = 25)	GP II (*N* = 25)	P2
Endoscopic assessment				
Nodule size (pixel)	Pre ttt	3.25 ± 0.94	2.79 ± 1.13	0.064
	1w post ttt	0.86 ± 0.63	1.07 ± 1.02	0.726
	1 m post ttt	0.73 ± 0.67	0.72 ± 0.73	0.814
	2 m post ttt	1.13 ± 0.93	0.68 ± 0.78	0.078
	3 m post ttt	1.57 ± 1.08	0.82 ± 1.14	0.003**
	P1	< 0.001**	< 0.001**	
Mucosal wave	Pre ttt	2.36 ± 0.49	2.6 ± 0.5	0.093
	1w post ttt	2.72 ± 0.46	3 ± 0	0.005**
	1 m post ttt	2.88 ± 0.33	3 ± 0	0.077
	2 m post ttt	2.72 ± 0.46	2.92 ± 0.28	0.068
	3 m post ttt	2.68 ± 0.48	2.92 ± 0.33	0.091
	P1	< 0.001**	< 0.001**	
Glottic gap	Pre ttt	1.48 ± 0.51	1.52 ± 0.51	0.779
	1w post ttt	2.2 ± 0.41	2.24 ± 0.66	0.641
	1 m post ttt	2.48 ± 0.51	2.48 ± 0.59	0.894
	2 m post ttt	2.04 ± 0.79	2.44 ± 0.65	0.067
	3 m post ttt	1.76 ± 0.66	2.4 ± 0.76	0.003**
	P1	< 0.001**	< 0.001**	
Subjective evaluation				
GRBAS	Pre ttt	7.48 ± 3.03	7.88 ± 3.22	0.639
	1w post ttt	3.32 ± 2.63	2.44 ± 2.29	0.257
	1 m post ttt	2.52 ± 2.31	1.4 ± 2.1	0.071
	2 m post ttt	3.16 ± 3.45	1.32 ± 2.14	0.048*
	3 m post ttt	3.96 ± 3.51	1.56 ± 2.47	0.010*
	P1	< 0.001**	< 0.001**	
VHI	Pre ttt	58.32 ± 21.28	53.84 ± 17.48	0.248
	1w post ttt	14.96 ± 21.07	12.8 ± 9.96	0.356
	1 m post ttt	9.52 ± 11.29	10.48 ± 8.11	0.312
	2 m post ttt	15.32 ± 18.67	10.36 ± 8.68	0.490
	3 m post ttt	23.96 ± 26.1	13.56 ± 16.12	0.244
	P1	< 0.001**	< 0.001**	
Objective evaluation				
Acoustics				
F0 (Hz)	Pre	208.42 ± 28.64	215.82 ± 23.79	0.594
	1w post ttt	221.65 ± 24.51	199.28 ± 15.75	0.001**
	1 m post ttt	211.67 ± 23.51	202.55 ± 14.00	0.093
	2 m post ttt	209.73 ± 20.56	202.41 ± 14.09	0.248
	3 m post ttt	210.91 ± 24.74	203.12 ± 13.88	0.528
	P1	0.006**	< 0.001**	
Jitter (%)	Pre	2.60 ± 1.50	2.37 ± 1.73	0.295
	1w post ttt	1.58 ± 0.47	1.21 ± 0.45	0.007**
	1 m post ttt	1.323 ± 0.644	1.160 ± 0.519	0.221
	2 m post ttt	1.48 ± 0.63	1.03 ± 0.38	0.007**
	3 m post ttt	1.601 ± 0.883	1.056 ± 0.477	0.016*
	P1	< 0.001**	< 0.001**	
Shimmer (dB)	Pre	2.60 ± 1.61	2.89 ± 1.62	0.426
	1w post ttt	2.56 ± 2.17	1.74 ± 1.12	0.535
	1 m post ttt	1.79 ± 1.65	1.58 ± 0.77	0.574
	2 m post ttt	1.88 ± 1.57	1.26 ± 1.04	0.130
	3 m post ttt	2.297 ± 1.734	1.361 ± 1.289	0.022*
	P1	< 0.001**	< 0.001**	
H/N (dB)	Pre	3.945 ± 4.590	4.949 ± 4.920	0.574
	1w post ttt	7.85 ± 2.83	8.72 ± 2.88	0.479
	1 m post ttt	9.5 ± 2.198	9.892 ± 2.706	0.560
	2 m post ttt	9.41 ± 2.94	10.55 ± 2.38	0.194
	3 m post ttt	7.64 ± 4.01	13.93 ± 15.49	0.001**
	P1	< 0.001**	< 0.001**	
Aerodynamic				
MPT (sec)	Pre	6.62 ± 1.40	6.97 ± 1.14	0.107
	1w post ttt	7.76 ± 2	8.72 ± 1.26	0.009**
	1 m post ttt	8.26 ± 1.58	9.1 ± 1.28	0.024*
	2 m post ttt	8.24 ± 1.76	9.44 ± 1.87	0.037*
	3 m post ttt	7.76 ± 1.85	9.62 ± 2.12	0.001**
	P1	< 0.001**	< 0.001**	

### Between-group differences

Nodule size was significantly lower at 3 months post-treatment in the voice therapy GP than in the steroid injection GP (P value = 0.003). The mucosal wave and glottic gap were markedly higher at 1 week (P value = 0.005) and 3 months (P value = 0.003) post-treatment, respectively, in the voice therapy GP than the steroid injection GP. GRBAS was lower at 2- and 3-months posttreatment in the voice therapy GP than the steroid injection GP (P value = 0.048 and 0.010, respectively). The total score of the VHI showed no statistically significant difference between both GPs across pretreatment and 1 week, 1-, 2, and 3-months posttreatment. F0 was significantly lower at 1-week post-treatment in the voice therapy GP than in the steroid injection GP (P value = 0.001). Jitter was significantly lower at 1 week and 2- and 3-months post-treatment in the voice therapy GP than the steroid injection GP (P value < 0.05). Shimmer and H/N were markedly lower (P value = 0.022) and higher (P value = 0.001) at 3-month post-treatment in the voice therapy GP than the steroid injection GP, respectively. MPT was significantly longer at 1 week and 1-, 2-, and 3-months post-treatment in the voice therapy GP than the steroid injection GP (P value < 0.05) (Table 2).

### Long-term follow-up (6 months)

At 6 months, all voice parameters except F0 and shimmer were significantly different (improved) in the steroid injection GP (P value < 0.05). While in the voice therapy GP, all voice parameters were significantly different (improved) at the sixth month (P value < 0.05).

Between groups, only the nodule size was significantly lower at 6 months post-treatment in the voice therapy GP than in the steroid injection GP (P value < 0.05). Most of the other subjective and objective voice parameters of the voice therapy GP (particularly glottic gap, GRBAS, shimmer, and MPT) performed better than the steroid injection GPs without reaching a significant level (Table 3).

**Table 3:** Comparison of subjective and objective voice parameters pre and 6 months post-treatment in both GPs

	GP I (*N* = 19)			GP II (*N* = 17)			P2	P3
	Pre ttt	Post 6 m	P1	Pre ttt	Post 6 m	P1		
Nodule size	3.37 ± 1.03	1.62 ± 1.04	< 0.001**	2.85 ± 1.21	0.97 ± 1.31	< 0.001**	0.093	0.049*
Mucosal wave	2.37 ± 0.5	2.79 ± 0.42	0.005**	2.53 ± 0.51	2.82 ± 0.39	0.025*	0.339	0.799
Glottic gap	1.47 ± 0.51	1.84 ± 0.6	0.008**	1.53 ± 0.51	2.29 ± 0.85	0.005 **	0.742	0.064
GRBAS	6.89 ± 2.94	3.47 ± 2.09	< 0.001**	7.76 ± 2.99	2.24 ± 3.31	< 0.001**	0.145	0.066
VHI	59.63 ± 22.25	20.11 ± 25.1	0.001**	53.59 ± 15.97	16.24 ± 19.01	< 0.001**	0.065	0.987
F0	208.11 ± 24.44	207.02 ± 25.45	0.809	215.22 ± 25.61	202.01 ± 18.96	0.031*	0.354	0.635
Jitter	2.53 ± 1.56	1.3 ± 0.54	0.003**	2.25 ± 1.63	1.19 ± 0.51	< 0.001**	0.739	0.447
Shimmer	2.85 ± 1.73	2.29 ± 1.81	0.112	2.62 ± 1.49	1.39 ± 1.37	0.006**	0.383	0.068
H/N	5.78 ± 3.81	8.91 ± 3.15	0.015*	6.22 ± 4.43	9.44 ± 3.08	0.019*	0.775	0.646
MPT	6.52 ± 1.42	7.78 ± 1.61	0.004**	7.09 ± 1.26	9.5 ± 2.43	< 0.001**	0.862	0.062

### Adverse effects of VFSI

None of the patients experienced severe side effects such as airway obstruction. However, 10 patients (40%) developed vocal fold hematomas, which were identified within the first week post-injection and resolved within one month. Additionally, a whitish deposition of triamcinolone acetonide occurred in 10 patients (40%) associated with minimal to moderate impairment of mucosal wave propagation. It was observed within 1 week after injection; 5 patients (20%) resolved at 1 month, while the remaining 5 patients (20%) disappeared within 2 months. Importantly, no patients experienced vocal fold atrophy during the follow-up periods.

**Fig. 3:**
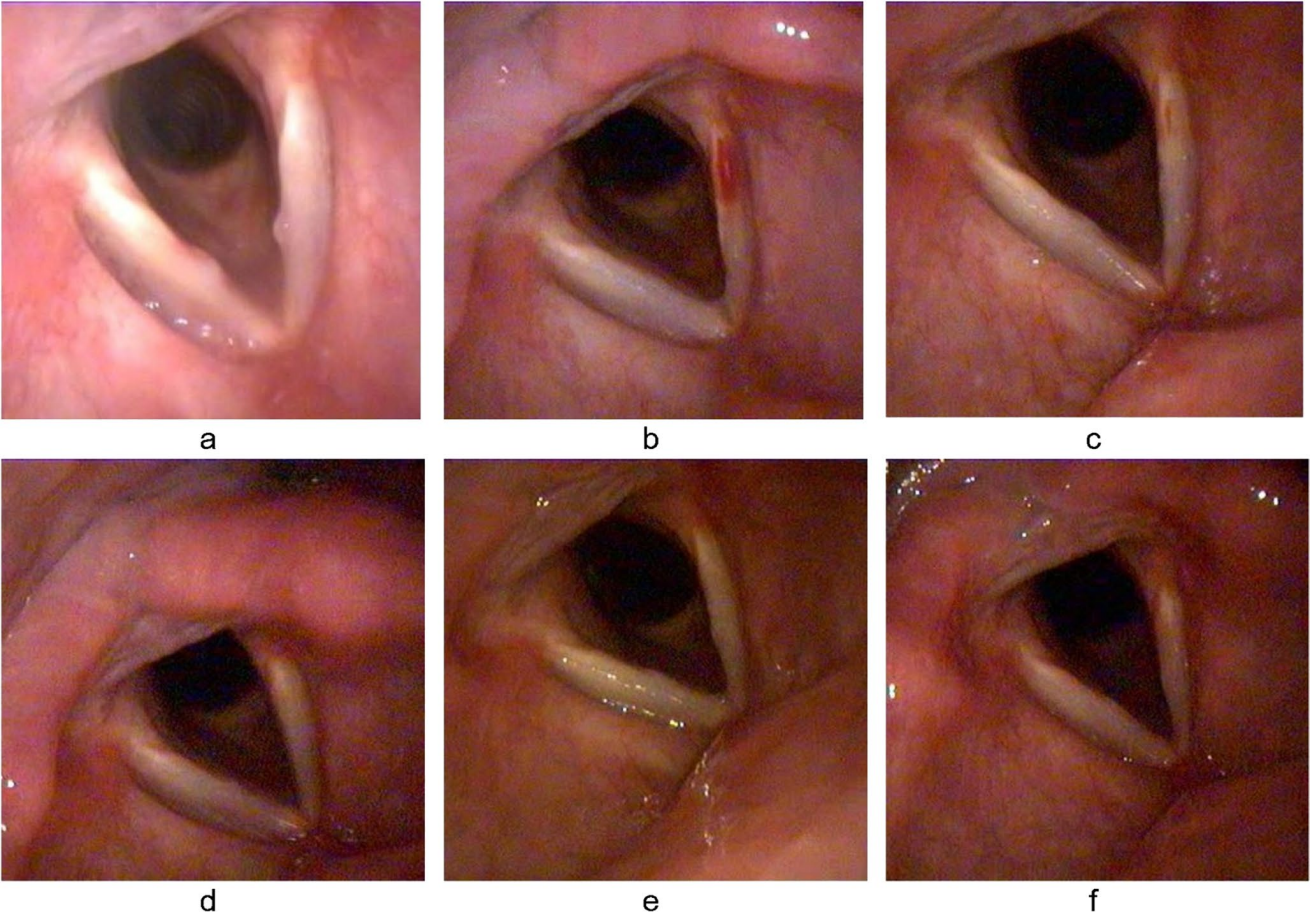
(**a**) Vocal nodules Pre-injection, (**b**) 1-week post-percutaneous injection of triamcinolone acetonide: submucosal hematoma at left VF with complete resolution of nodules; (**c**) 1-month post-injection: with resolution of submucosal hematoma; (**d**) 2 months post-injection; (**e**) 3 months post-injection: beginning of nodule recurrence ; (**f**) 6 months post-injection

**Fig. 4:**
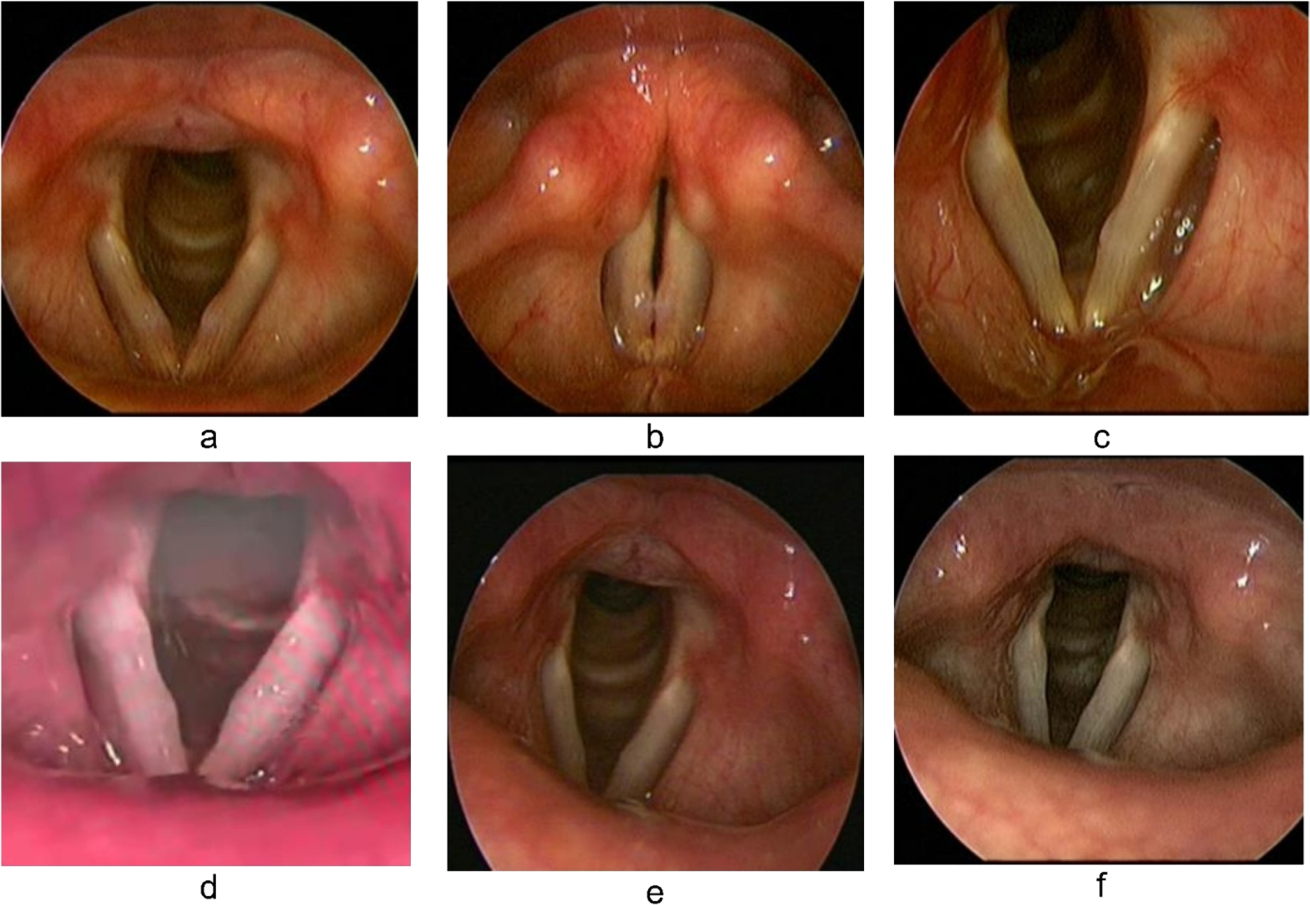
(**a**) Vocal nodules Pre-AM voice therapy: during respiration. Note the increased vascular markings of VF; (**b**) during phonation. (**c**) 1-week post-AM voice therapy: resolution of VF redness with partial resolution of nodules; (**d**) 1-month post-AM voice therapy; (**e**) 2 months post-AM voice therapy; (**f**) 3 months post-AM voice therapy: complete resolution of nodules

Figures 3 and 4 show serial VLS images of vocal nodules before and through the follow up visits after VFSI and voice therapy, respectively.

## Discussion

VFN treatment primarily relies on voice therapy, but its lengthy duration and multiple sessions may hinder patient compliance due to economic or occupational factors. Steroids treat VFNs resulting from phonotrauma-induced edema, inflammation, and damaged epithelial barriers [33]. Injecting them intralesionally offers the advantage of preserving the localized therapeutic effect of corticosteroids while minimizing systemic or adverse side effects [19]. Although many studies have evaluated the efficacy of VFSI in VFNs and other benign vocal fold lesions by comparing pre- and post-treatment measures [12, 16, 20, 21, 34, 35], the absence of a control GP receiving the standard treatment may overestimate its effectiveness. Consequently, the principal and secondary goals of the present research were to evaluate the multidimensional treatment outcomes at 6 months after intervention in patients undergoing either VFSI or AM voice therapy and directly compare the treatment outcomes between the two GPs.

Patient selection focused on females, given their higher vocal nodule prevalence [1], and specifically on soft edematous nodules due to evidence supporting their responsiveness to the intended therapies [18, 27]. Methodological choices prioritized established benefits: the trans-cricothyroid steroid injection offered technical accessibility and familiarity, utilizing triamcinolone acetonide for its potent anti-edema action, local retention, and sustained effects [13]. For the control GP, Smith Accent therapy provided a holistic intervention; its rhythmic abdominal exercises are proposed to rebalance laryngeal physiology by improving pulmonary support, thereby potentially augmenting the Bernoulli effect at the glottis [27].

In the current study, both treatment GPs demonstrated significant reductions in nodule size at the 3-month follow-up. This finding aligns with prior transnasal endoscopic steroid injection (TESI) studies, including one reporting an 81.25% decrease in nodule size at 3 weeks [26] and another demonstrating 31% complete resolution and 69% significant improvement in VFN patients at 3 months [19]. However, when comparing both GPs, the size of nodules increased markedly in the steroid injection GP relative to the voice therapy GP at the third month post-treatment. This observation suggests potential recurrence or suboptimal durability of the VFSI effect, consistent with reports of nodule relapse (defined as recurrence or size increase after initial response) when follow-up extends beyond 1 month post-injection [12, 18, 24]. This recurrence addresses the primary cause of vocal nodules, which is voice misuse and abuse, and they may recur until the vocal misuse and overuse habits are altered [4, 36–38].

A significant improvement in mucosal wave was observed within both GPs by the 3-month follow-ups. This agreed with Lee and Park, who noted that the mucosal wave was significantly increased at three months post-percutaneous steroid injection [24]. Notably, the steroid injection GP demonstrated a marked decrease in the mucosal wave at 1week post-treatment compared to the voice therapy GP. This could be explained by the thick plaque formation of the injected triamcinolone [18].While both GPs ultimately improved, the early differential effect highlights a transient disadvantage of injection.

Significant improvement in glottic gap was reported in both GPs by the 3-month follow-up, again matching the results of Lee and Park [24]. However, when comparing the two studied GPs, the voice therapy GP showed a marked improvement in glottic gap compared to the steroid injection GP at the third month post-treatment. This finding indicates better glottal behavior by AM, while the steroid injection GP experienced recurrence.

As regards GRBAS, it was significantly decreased (i.e., better) during the 3 months of post-treatment in both GPs. This finding agrees with studies reporting significant reduction in the grade of dysphonia within 3 weeks [26] up to 3 months [19] post-TESI. Nevertheless, the GRBAS scores were markedly increased in the steroid injection GP relative to the voice therapy GP at 2- and 3-months follow-ups. Wang et al. found that GRB scores of VFN patients significantly improved after 1 and 2 months post steroid injection with the initial month showing the most significant improvement [18].

Total scores of the VHI demonstrated a marked decrease (indicating improved quality of life) in the 3 months post-treatment within both GPs. This result is encouraged by Elsaeed et al., who reported that the VHI improved significantly after TESI for VFNs, comparable to outcomes achieved with phonomicrosurgery [26]. Notably, there was no statistically marked difference in VHI scores among the two GPs either before or 3 months after treatment.

Divergent F0 results were observed among the treatment GPs. In the steroid injection GP, the F0 was markedly higher compared to pretreatment levels throughout the entire 3 months post-treatment. Conversely, the voice therapy GP reported a marked reduction in F0 over the same follow-up period. Consequently, a direct comparison revealed markedly higher F0 values in the steroid injection GP relative to the voice therapy GP at 1-week post-treatment. We hypothesized that increased F0 post VFSI could result from reduced VFN edema and inflammation, leading to decreased VF mass and improved viscoelastic properties [39]. Whereas, the reduction in F0 post voice therapy is consistent with reduced laryngeal muscle tension, improved glottic efficiency, and reduced subglottic pressure, which are therapeutic targets in managing hyperfunctional element often seen in nodules [8, 40]. It is important to note that despite these contradictory results within GPs, no significant differences between GPs in F0 were detected before or during the follow-up period, with the exception of the initial 1 week. This transient increase in F0 after injection was hypothesized to be a behavioral adaptation to the temporary stiffness and vibratory impairment of the vocal folds caused by hematoma, which eventually resolved [41]. The pattern of increased F0 post steroid injection is reported by Takahashi et al. and Nozawa et al. [17, 42]. They documented a marked improvement in the fundamental speech frequency 2–3 and 3–4 months post-VFSI, respectively.

Regarding other acoustic objective voice parameters, we observed significant improvements across key perturbation and noise measures within both GPs over the 3-month period. Jitter and shimmer decreased significantly, while the H/N ratio increased significantly. The enhancement of the acoustic measurements can be accounted for by enhanced vocal folds’ contact and better vibratory patterns. Lee and Park et al. supported our results, as they demonstrated significant improvement of jitter, shimmer, and H/N ratio post three months of VFSI [24]. However, comparative analysis revealed distinct outcomes between-GPs: jitters were markedly elevated in the steroid injection GP relative to the voice therapy GP at 1 week and 2- and 3-months post-treatment. Additionally, by the third month, shimmer was significantly higher, and the H/N ratio was markedly reduced in the steroid injection relative to the voice therapy GP, reflecting nodule recurrence.

MPT was significantly increased from baseline over the entire three months post-treatment in both studied GPs. This improvement is likely to contribute to reduced nodule size and glottal resistance in both GPs. Our results report an increase in MPT post-VFSI align with several studies demonstrating significant improvement for VFN patients 2 and 3 months post-VFSI [17–19]. However, MPT was consistently and significantly longer in the voice therapy GP compared to the steroid injection GP at every post-treatment follow-up point. The superiority of MPT in voice therapy may be obtained from improved glottal closure, pulmonary support, and laryngeal muscle tone. While the lower MPT in VFSI may be due to inadequate respiratory strategies or subclinical atrophic alteration in certain patients.

At the six-month follow-up, the steroid injection group maintained statistically significant improvements in the majority of subjective and objective voice measures compared to pretreatment baselines. These findings align with Woo et al., who reported significant improvement in the mucosal wave, glottic gap, VHI, and jitter post-6 m VFSI follow-up [20]. Conversely, the voice therapy group demonstrated significant improvements across all subjective and objective voice measures compared to pretreatment. In general, there aren’t many published long-term voice therapy studies, yet evidence suggests sustained efficacy. Fu et al. documented persistent improvements in patient self-perception, perceptual, stroboscopic, and acoustic voice measurements 6 months post-therapy [43]. Direct comparison revealed significantly smaller nodule size in the voice therapy group versus the steroid injection group, suggesting potentially greater efficacy in reducing nodule recurrence. Furthermore, most of the other voice parameters favored the voice therapy group, although these differences lacked statistical significance. On the other hand, the injection cohort showed unexpected improvement in almost all voice parameters at six months, despite a prior decline at three months. The interpretation of both GPs are most plausibly attributed to attrition bias caused by loss to follow-up, as participant counts fell from 25 to 19 in GP I and from 25 to 17 in GP II. Regarding nodule recurrence, Lee and Park reported a 26.7% nodule recurrence within average 8.5 months post-VFSI [22]. In contrast, Bakat et al. observed recurrence in only 1 of 18 VFN patients 6 months post-voice therapy [44].

Regarding post-injection adverse events, our study views the transient deterioration of both the mucosal wave and some accompanying acoustic parameters as a temporary consequence of procedural hematoma—which affected 40% of the injection cohort and likely contributed to the one-week group differences—rather than as a direct steroid effect. Prior research has shown variable rates of VF hematoma post-VFSI. Woo et al. and Lee and Park reported that it occurred in a small percentage of patients, 1.6% and 2.4%, respectively, and cleared spontaneously [20]. The discrepancy between those results and ours could be attributed to their larger sample size, longer first post-injection follow-up period, or the utilization of percutaneous injection techniques other than the transcricothyroid (which makes the injection needle more visible). Meanwhile, other VF benign lesion steroid injection studies showed a higher percentage of hematoma: 27% [18] up to most of the studied patients (*n* = 40) [17], which resolved within 1 month. Wang et al. attributed their elevated rate primarily to the transoral and transnasal injection techniques employed, which required direct puncture through the vocal fold epithelium [18]. They also identified pre-existing vocal fold varices and high occupational vocal demand as significant risk factors for postoperative hematoma. Additionally, studies show a wide incidence range of triamcinolone whitish deposition (2.5–100%) [20, 21, 45], with earlier clinic visits resulting in more side effect detection, indicating that triamcinolone is absorbed gradually.

The limitations of the study include insufficient sample size for robust subgroup analysis, a relatively brief follow-up duration, a lack of blinding owing to distinct interventions, and not examining larger nodule sizes and their effect on treatment response. The interpretation of the post-VFSI outcomes is initially hindered by a 40% rate of procedural hematomas, a confounding factor, and also compromised by substantial participant dropout at the six-month follow-up.

## Conclusions

Office-based percutaneous VF triamcinolone injection is a rapid, safe, well-tolerated, and effective therapy for VFNs with outcomes comparable to voice therapy. Long-term data, however, demonstrate voice therapy better prevents nodule recurrence. Therefore, subsequent voice therapy or repeated injections may enhance the long-term efficacy of VFSI and reduce nodule recurrence.

## Data Availability

Data is available on reasonable requests from the corresponding author.

## References

[1] Verdolini K, Rosen CA, Branski RC (2014) Classification manual for voice disorders-ⅰ, vol 07430. Psychology, Mahwah, New Jersey

[2] Van Houtte E, Van Lierde K, D’haeseleer E, Claeys S (2010) The prevalence of laryngeal pathology in a treatment-seeking population with dysphonia. Laryngoscope 120:306–312. 10.1002/lary.20696 19957345 10.1002/lary.20696

[3] Glanz H, Schulz A, Kleinsasser O, Schulze W, Dreyer T, Arens C (1997) Benign lesions of the larynx: basic clinical and histopathological data. In: Kleinsasser O, Glanz H, Olofsson J (eds) Advances of Laryngology in Europe. Elsevier, Amsterdam, pp 3–14

[4] Wallis L, Jackson-Menaldi C, Holland W, Giraldo A (2004) Vocal fold nodule vs. Vocal fold polyp: answer from surgical pathologist and voice pathologist point of view. J Voice 18:125–129. 10.1016/j.jvoice.2003.07.003 15070232 10.1016/j.jvoice.2003.07.003

[5] Aronson A, Bless D (2009) Voice disorders of structural origin. In: Aronson A, Bless D (eds) Clinical voice disorders. Thieme Med Pub, New York, pp 24–38

[6] Aronsson C, Bohman M, Ternström S, Södersten M (2007) Loud voice during environmental noise exposure in patients with vocal nodules. Logopedics Phoniatrics Vocology 32:60–70. 10.1080/14015430601002408 17613787 10.1080/14015430601002408

[7] Casper JK, Leonard R (2006) Understanding voice problems: a physiological perspective for diagnosis and treatment. Lippincott Williams & Wilkins

[8] Holmberg EB, Doyle P, Perkell JS, Hammarberg B, Hillman RE (2003) Aerodynamic and acoustic voice measurements of patients with vocal nodules: variation in baseline and changes across voice therapy. J Voice 17:269–282. 10.1067/S0892-1997(03)00076-6 14513951 10.1067/s0892-1997(03)00076-6

[9] Verdolini K, Ramig LO (2001) Occupational risks for voice problems. Logopedics Phoniatrics Vocology 26:37–46. 10.1080/14015430119969 11432413

[10] Bassiouny S (1998) Efficacy of the accent method of voice therapy. Folia Phoniatr Et Logopaedica 50:146–164. 10.1159/000021458 10.1159/000021458 9691529

[11] Tateya I (2009) Laryngeal steroid injection. Current opinion in otolaryngology &. Head Neck Surg 17:424–426. 10.1097/MOO.0b013e3283327d4c 10.1097/MOO.0b013e3283327d4c 19779349

[12] Tateya I, Omori K, Kojima H, Hirano S, Kaneko K-i, Ito J (2004) Steroid injection to vocal nodules using fiberoptic laryngeal surgery under topical anesthesia. Eur Archives Oto-Rhino-Laryngology Head Neck 261:489–492. 10.1007/s00405-003-0720-x 10.1007/s00405-003-0720-x 15546175

[13] Benjamin B (1998) Vocal nodules. In: Benjamin B (ed) Endolaryngeal surgery Dunitz. CRC, Dunitz, London, pp 113–124

[14] Campagnolo AM, Tsuji DH, Sennes LU, Imamura R, Saldiva PH (2010) Histologic study of acute vocal fold wound healing after corticosteroid injection in a rabbit model. Annals Otology Rhinology Laryngology 119:133–139. 10.1177/000348941011900211 10.1177/000348941011900211 20336925

[15] Baraka M, Behairy EA-e, El Desouky H, Mostafa S, Ezzat E (2021) Office-based steroid injection in benign vocal fold lesions. Egypt J Ear Nose Throat Allied Sci 22:1–9. 10.21608/ejentas.2021.86930.1396

[16] Mortensen M, Woo P (2006) Office steroid injections of the larynx. Laryngoscope 116:1735–1739. 10.1097/01.mlg.0000231455.19183.8c 17003727 10.1097/01.mlg.0000231455.19183.8c

[17] Takahashi S, Kanazawa T, Hasegawa T, Hirosaki M, Komazawa D, Konomi U, Nimura Y, Sakaguchi Y, Nozawa M, Yamauchi T (2021) Comparison of therapeutic effects of steroid injection by benign vocal fold lesion type. Acta Otolaryngol 141:1005–1013. 10.1080/00016489.2021.1995895 34751085 10.1080/00016489.2021.1995895

[18] Wang C-T, Lai M-S, Hsiao T-Y (2015) Comprehensive outcome researches of intralesional steroid injection on benign vocal fold lesions. J Voice 29:578–587. 10.1016/j.jvoice.2014.11.002 25944294 10.1016/j.jvoice.2014.11.002

[19] Wang CT, Lai MS, Liao LJ, Lo WC, Cheng PW (2013) Transnasal endoscopic steroid injection: A practical and effective alternative treatment for benign vocal fold disorders. Laryngoscope 123:1464–1468. 10.1002/lary.23715 23494523 10.1002/lary.23715

[20] Woo J-H, Kim D-Y, Kim J-W, Oh E-A, Lee S-W (2011) Efficacy of percutaneous vocal fold injections for benign laryngeal lesions: prospective multicenter study. Acta Otolaryngol 131:1326–1332. 10.3109/00016489.2011.620620 22074107 10.3109/00016489.2011.620620

[21] Lee S-H, Yeo J-O, Choi J-I, Jin H-J, Kim J-P, Woo S-H, Jin S-M (2011) Local steroid injection via the cricothyroid membrane in patients with a vocal nodule. Archives Otolaryngology–Head Neck Surg 137:1011–1016. 10.1001/archoto.2011.168 10.1001/archoto.2011.168 22006779

[22] Wang C-T, Lai M-S, Cheng P-W (2017) Long-term surveillance following intralesional steroid injection for benign vocal fold lesions. JAMA Otolaryngology–Head Neck Surg 143:589–594. 10.1001/jamaoto.2016.4418 10.1001/jamaoto.2016.4418 PMC5824226 28334309

[23] Wang CT, Liao LJ, Lai MS, Cheng PW (2014) Comparison of benign lesion regression following vocal fold steroid injection and vocal hygiene education. Laryngoscope 124:510–515. 10.1002/lary.24328 23908142 10.1002/lary.24328

[24] Lee SW, Park KN (2016) Long-term efficacy of percutaneous steroid injection for treating benign vocal fold lesions: A prospective study. Laryngoscope 126:2315–2319. 10.1002/lary.25916 26971530 10.1002/lary.25916

[25] Ramavat AS, Tiwana H, Banumathy N, Bakshi J, Panda N, Goel A (2019) Efficacy of intralesional steroid injection in small benign vocal fold lesions. J Voice 33:767–772. 10.1016/j.jvoice.2018.04.008 30077419 10.1016/j.jvoice.2018.04.008

[26] Elsaeed A, Afsah O, Nawka T, Caffier P, Baz H (2023) Treatment of vocal fold nodules: transnasal steroid injection versus microlaryngoscopic phonomicrosurgery. J Voice. 10.1016/j.jvoice.2023.02.003 36882331 10.1016/j.jvoice.2023.02.003

[27] Kotby M, El-Sady S, Basiouny S, Abou-Rass Y, Hegazi M (1991) Efficacy of the accent method of voice therapy. J Voice 5:316–320. 10.1016/S0892-1997(05)80062-1

[28] Hirano M (1981) Clinical examination of voice. Springer-, New York

[29] Malki KH, Mesallam TA, Farahat M, Bukhari M, Murry T (2010) Validation and cultural modification of Arabic voice handicap index. Eur Arch Otorhinolaryngol 267:1743–1751. 10.1007/s00405-010-1296-x 20532904 10.1007/s00405-010-1296-x

[30] Mallur PS, Tajudeen BA, Aaronson N, Branski RC, Amin MR (2011) Quantification of benign lesion regression as a function of 532-nm pulsed potassium Titanyl phosphate laser parameter selection. Laryngoscope 121:590–595. 10.1002/lary.21354 21298636 10.1002/lary.21354

[31] Mallur PS, Rosen CA (2010) Vocal fold injection: review of indications, techniques, and materials for augmentation. Clin Exp Otorhinolaryngol 3:177–182. 10.3342/ceo.2010.3.4.177 21217957 10.3342/ceo.2010.3.4.177 PMC3010535

[32] Kotby MN (1995) The accent method of voice therapy. Singular Publishing Group, Inc., San Diego, California

[33] Wang C-T (2021) Vocal fold steroid injection. In: Lee B-J, Kwon T-K, Rosen CA (eds) Vocal fold injection. Springer, Singapore, p 141–149

[34] Hsu Y-B, Lan M-C, Chang S-Y (2009) Percutaneous corticosteroid injection for vocal fold polyp. Archives Otolaryngology–Head Neck Surg 135:776–780. 10.1001/archoto.2009.86 10.1001/archoto.2009.86 19687397

[35] Tateya I, Omori K, Kojima H, Hirano S, Kaneko K, Ito J (2003) Steroid injection for reinke’s edema using fiberoptic laryngeal surgery. Acta Otolaryngol 123:417–420. 10.1080/00016480310001321 12737301 10.1080/00016480310001321

[36] Altman KW (2007) Vocal fold masses. Otolaryngol Clin North Am 40:1091–1108 17765697 10.1016/j.otc.2007.05.011

[37] Holmberg EB, Hillman RE, Hammarberg B, Södersten M, Doyle P (2001) Efficacy of a behaviorally based voice therapy protocol for vocal nodules. J Voice 15:395–412. 10.1016/S0892-1997(01)00041-8 11575636 10.1016/S0892-1997(01)00041-8

[38] Murry T, Woodson GE (1992) A comparison of three methods for the management of vocal fold nodules. J Voice 6:271–276. 10.1016/S0892-1997(05)80153-5

[39] Campagnolo AM, Tsuji DH, Sennes LU, Imamura R (2008) Steroid injection in chronic inflammatory vocal fold disorders, literature review. Revista Brasileira De Otorrinolaringologia 74:926–932. 10.1590/S0034-72992008000600017 19582351 10.1016/S1808-8694(15)30155-5 PMC9445967

[40] Fex B, Fex S, Shiromoto O, Hirano M (1994) Acoustic analysis of functional dysphonia: before and after voice therapy (accent method). J Voice 8:163–167. 10.1016/S0892-1997(05)80308-X 8061772 10.1016/s0892-1997(05)80308-x

[41] Zhang Z (2017) Effect of vocal fold stiffness on voice production in a three-dimensional body-cover phonation model. J Acoust Soc Am 142:2311–2321. 10.1121/1.5008497 29092586 10.1121/1.5008497 PMC5654985

[42] Nozawa M, Kanazawa T, Kurakami K, Kashima K, Okui A, Hasegawa T, Hirosaki M, Kamitomai M, Igarashi T, Ito M (2023) Age-dependent treatment effect of vocal fold steroid injection for benign vocal fold lesions. Laryngoscope Invest Otolaryngol 8:177–184. 10.1002/lio2.997 10.1002/lio2.997 PMC9948573 36846402

[43] Fu S, Theodoros D, Ward EC (2016) Long-term effects of an intensive voice treatment for vocal fold nodules. Int J Speech Lang Pathol 18:77–88. 10.3109/17549507.2015.1081286 28425364 10.3109/17549507.2015.1081286

[44] Bakat B, Gupta A, Roy A, Roychoudhury A, Raychaudhuri BK (2014) Does voice therapy cure all vocal fold nodules? Int J Phonosurgery Laryngology 4:55–59. 10.5005/jp-journals-10023-1083

[45] Filho PAA, Rosen CA (2003) Vocal fold plaque following triamcinolone injection. Ear Nose Throat J 82:908–911. 10.1177/014556130308201204 14702869

